# Measuring Quality in Emergency Medical Services: A Review of Clinical Performance Indicators

**DOI:** 10.1155/2012/161630

**Published:** 2011-10-15

**Authors:** Mazen J. El Sayed

**Affiliations:** EMS and Prehospital Care Program, Department of Emergency Medicine, American University of Beirut Medical Center, P.O. Box 11-0236, Riad El Solh, Beirut 110 72020, Lebanon

## Abstract

Measuring quality in Emergency Medical Services (EMSs) systems is challenging. This paper reviews the current approaches to measuring quality in health care and EMS with a focus on currently used clinical performance indicators in EMS systems (US and international systems). The different types of performance indicators, the advantages and limitations of each type, and the evidence-based prehospital clinical bundles are discussed. This paper aims at introducing emergency physicians and health care providers to quality initiatives in EMS and serves as a reference for tools that EMS medical directors can use to launch new or modify existing quality control programs in their systems.

## 1. Background

Measuring quality in emergency medical services (EMSs) is important since EMS is the practice of medicine in the prehospital setting. At its earliest developmental stage, an EMS system is a system of emergency care that functions to reduce death and disability usually resulting from two epidemics: trauma and cardiac arrest. In the United States, EMS systems have witnessed a major transformation since the EMS program was first established in 1966 in the Department of Transportation through the Highway Safety Act. The expansion in EMS scope and increase in the range of medical interventions performed by prehospital providers were paralleled with an increased scrutiny of the value and effectiveness of the services deployed in the prehospital setting [1, 2]. The need for increased coordination in patient care and higher quality care at lower costs has made it essential for EMS agencies to have in-place quality control or quality improvement programs that rely on key performance indicators to continuously monitor the system's overall performance and the effectiveness of the different prehospital interventions. 

The Institute of Medicine (IOM), in a report entitled “Emergency Medical Services at the Crossroads” and published in 2006, recommended the development of “evidence based performance indicators that can be nationally standardized so that statewide and national comparisons can be made” [3]. The development and implementation of these indicators would enhance accountability in EMS and provide EMS agencies with data to measure their system's overall performance and to develop sound strategic quality improvement planning. The objective of this paper is to introduce emergency physicians and other healthcare providers to the concepts of quality measurement in EMS with a focus on clinical performance indicators currently used by EMS agencies in the USA and by ambulance services in other countries such as the United Kingdom and Australia.

## 2. Quality Care in EMS: Definition and Challenges

A central premise is that the same principles of healthcare quality apply to EMS. Many definitions of quality in health care exist (Donabedian and the American Medical Association definitions of quality) [3, 4]; however the most widely cited one and most applicable to EMS systems is the definition formulated by the IOM. The IOM defined quality as “the degree to which health services for individuals and populations increase the likelihood of desired health outcomes and are consistent with current professional knowledge” and described six dimensions of quality care: a care that is safe, effective, patient centered, timely, efficient, and equitable [5]. When applied to EMS, the IOM concepts on quality care therefore entail a system design with a specific arrangement of personnel, facilities, and equipment that functions to ensure not only effective and coordinated delivery of health care services under emergency conditions but also high quality appropriate care. This ideal system design is nonexistent since most EMS systems evolved as a response to the communities' needs for emergent health care services (military conflicts, major highways trauma, and nontraumatic cardiac arrest) rather than as an a priori designed EMS infrastructure. This resulted in heterogeneity of existing EMS systems designs [6, 7] making EMS systems complex and difficult to evaluate or compare.

Other EMS-specific challenges to systems evaluation include the lack of uniformity in data collection and the lack of agreement over the validity of the performance indicators or assessment measures used in EMS research [8, 9]. Adding to that, the existence of a broad range of EMS conditions (i.e., conditions that cause EMS activation) and the challenge of isolating the prehospital care effect from that of the emergency department and hospital care increase the complexity of measuring quality in EMS [10, 11].

## 3. Approaches to Quality Measurement

Initiatives to incorporate quality assessment in EMS, similar to other healthcare settings, have adopted the frameworks and principles of quality management systems used in the business industry. The goal is to improve the end product and the customer's satisfaction. In EMS, the end product is the care provided to patients in the prehospital setting. Quality programs usually range from basic traditional Quality Assurance (QA) to Continuous Quality Improvement (CQI) and complex Total Quality Management (TQM). Quality assurance is the static and retrospective review or inspection of services to identify problem areas [12, 13]. Quality improvement requires a continuous study and improvement of a process, system, or organization [13]. Total Quality Management is the most advanced and most comprehensive since it involves the whole organization. Elements of TQM consist of leadership commitment to quality, use of reliable indicators of quality, and involvement of front-line workers in quality improvement efforts [12]. The shift in quality management paradigm in EMS from pure quality assurance programs towards quality improvement took place after the adoption of CQI concept by the Joint Commission on Accreditation of Healthcare Organizations (JCAHO) in 1992. EMS quality assessment focused more on improving patient care through continuous measurements and inputs using key performance indicators [14].

## 4. EMS System Performance Indicators: Structure-Process-Outcome Model

Performance indicators are measurement tools that should be “specific, measurable, action oriented, relevant and timely” [15]. Three types of indicators are used to measure quality in patient care: Structure, process and outcome indicators (Table 1) [16–19]. EMS system performance indicators follow the same classification. 

Structural data are attributes of the setting in which care is provided [17]. These usually refer to the characteristics of the different components of an EMS system including facilities, equipment, staffing, knowledge base of providers, credentialing, deployment. Most structure indicators reflect standards developed at a local, regional, or national level through consensus building or by EMS organizations, administrators, or authority. These indicators provide an indirect measure of quality and are difficult to relate to outcomes in patient care [19]. Since EMS systems designs are diverse as discussed above, these indicators may not be applicable to all systems. Emergency vehicle response time standard is the most commonly used structure measure in EMS. The goal is to respond to 90% of priority 1 calls (life threatening and highly time dependant) in less than 9 minutes [22]. Several EMS systems designed ambulance deployment strategies to meet this standard despite conflicting results from several studies about the effect of short response times on patient outcome in trauma [23–25] and the need for even shorter EMS times (around 4 minutes) to impact survival in out-of-hospital cardiac arrest [26, 27]. In the United Kingdom, the adoption of a similar time target structure measure (8-minute response time for 75% of category A or emergency calls) by the National Service Framework for coronary artery disease as the main performance indicator was criticized and described by the paramedics as a poor quality indicator that is “too simplistic and narrow” and that is putting patients and ambulance crews at risk [28]. 

Another type of measures is process data. These are the components of the encounter between the prehospital provider and the patient. It is an evaluation of the steps of the care provided. A process is the repeatable sequence of actions used across all EMS levels to produce good patient outcome [19]. Process measures are more sensitive to differences in quality of care [29]. In contrast to structure and outcome measures that provide an indirect approach to quality measurement, process measures allow for a direct assessment of quality of care. The inputs from process measures are very useful for quality improvement programs since they are easy to interpret and act on [30, 31]. One disadvantage of using process measures to monitor quality is that they can become very complex with increased clinical sophistication of the medical services provided in the prehospital setting. In the USA, EMS medical directors commonly use process measures when performing a structured implicit review of prehospital records (run sheets) to evaluate compliance with medical protocols and appropriateness of the treatment provided. One example would be collecting specific data points on the process of endotracheal intubation performed by EMS providers to monitor the success rate of this procedure. A medical director can evaluate the technical skill of providers performing this procedure and their compliance with preestablished criteria and decide which specific elements will need improvement such as mandating tube placement verification with End Tidal CO2 waveform documentation. 

A third type of measures is outcome data. These evaluate the change in patient's subsequent health status in response to a clinical intervention. Numerous prehospital interventions are not yet evidence based [2, 31–33]. Outcome research in EMS focuses on determining the effectiveness of some of these interventions and showing the true value of an EMS system since it offers feedback on all aspects of care. Outcome data is easy to interpret and easily understood by the different stakeholders (policymakers, patients, EMS providers, public, etc.) and can be used to compare EMS systems. The adoption of pure outcome data as performance indicators is not however straightforward. For outcome data and more specifically clinical outcome data, to be relevant performance indicators, accurate risk adjustment, standardization of definitions, and development of research models for each measured outcome are required [11, 29, 30]. Four explanations for the source of variation in outcome were described by Mant: these include differences in type of patient (patient characteristics), differences in measurement, chance (random variation), and differences in quality of care [30]. Adding to these challenges are the degree of sophistication of some prehospital treatment technologies, the operational complexity of the prehospital environment, and the difficulty in isolating the prehospital effect from the emergency department and hospital effect [11]. In an effort to overcome the barriers to outcome research and the adoption of outcome data as performance indicators for EMS systems, the US National Highway Traffic Safety Administration (NHTSA) launched in 1994 the EMS Outcomes Project (EMSOP) [10]. This project identified the priority conditions that should take precedence in EMS outcomes research based on impact and frequency and defined six outcome categories (6D's): survival (death), impaired physiology (disease), limit disability (disability), alleviate discomfort (discomfort), satisfaction (dissatisfaction), and cost-effectiveness (destitution) [10]. Two framework models were also proposed to facilitate outcome measurement in EMS: the “Episode of Care Model” and the “Out-of-Hospital Unit of Service Model” [11]. The first model is used for high-priority conditions (highly time dependent) to measure long-term outcomes (survival, physiologic derangement, long term disability). The second one is used for low-priority conditions (minor trauma) to measure short- or intermediate-term outcomes (patient satisfaction, relief of pain). Examples of core risk adjustment measures (RAMS) that are common to all EMS conditions (e.g., age, gender and vital signs) and specific RAMS (e.g., peak flow measurement for asthma exacerbation) were also described [34]. All these steps were designed to facilitate outcome research and the adoption of outcome measures in evaluating quality in EMS.

 Internationally, out-of-hospital cardiac arrest (OHCA) survival is the most common outcome measure used to compare EMS systems. Standardized risk adjustment measures and data collection forms are well defined (Utstein template) [35]. The incorporation of these elements in quality programs would enable EMS administrators and medical directors to compare outcomes with other systems, to identify the specific components of the system that are functioning properly and those that are not and how changes can be implemented to improve cardiac arrest outcomes.

## 5. Transitioning from Theory to Practice

Relying on only one type of performance measures (structure, process, or outcome) can yield a very narrow perspective on quality care in EMS. The complexity of EMS systems requires a more comprehensive evaluation of the different components of the system. 

One approach is to use a set of mixed indicators that cover different aspects of an EMS system. Several EMS stakeholders in the USA have proposed comprehensive sets of indicators: one set was proposed by the International Association of Firefighters in the National Fire Protection Association (NFPA) in several publications on standards of emergency medical operations (NFPA 1710), criteria for response times (NFPA 1720), and dispatch standards (NFPA 1221) [36]. Another set of 35 consensus-based indicators was proposed by the National Association of EMS Officials (NAEMSO) in 2007 at the end of the EMS Performance Measures Project in an effort to identify a common set of specifically defined measures of EMS system performance [37]. A tool was also proposed for EMS agencies to properly measure these indicators. Other sets of indicators are used by international ambulance services such as those used by the South Australian ambulance services and which are part of performance framework encompassing “operational, managerial and strategic level” indicators [38]. The validity and practical application of these indicators are yet to be tested. 

Another approach is to focus on few high impact clinical conditions and to use bundles of measures that are disease or condition specific in order to evaluate quality in the overall system. Evaluating the system's response to few high-priority clinical conditions considered “tracer conditions” can help predict performance of same elements in response to other clinical entities [39–41]. “Tracer conditions” such as trauma or cardiac arrest are clinical entities with high impact (mortality and morbidity, cost and frequency) and potential for improved outcome [10, 40, 42]. The bundles of measures are similar to composite measures that link structure and process to outcome. The elements of the bundle are evidence-based measures that would lead to improved patient outcome when combined together. One example of evidence-based treatment bundles is the set of clinical performance indicators proposed in 2007 by the US Consortium of Metropolitan Municipalities EMS directors [20]. Six EMS priority conditions were selected based on available supporting evidence of an effective prehospital treatment and on a consensus of EMS experts. Specific outcomes were described in the form of number needed to treat (NNT) and the harm avoided if the bundle measures were met in each case. A similar approach was used by the UK Care Quality Commission in proposing a different set of evidence-based indicators following the recommendations of Joint Royal Colleges Ambulance Liaison Committee (JRCALC) in 2006 [21]. Comparing the US and UK sets reveals overlap between some of the clinical conditions and the indicators that are proposed (Table 2). The outcomes that were defined by the UK set were however less specific than those of the US set. The goal of these bundles, when used in combination with standardized outcome categories, is to establish evidence-based benchmarks or best practices for EMS systems or ambulances services and to allow comparison of performance between different systems [20, 43]. Prerequisites for the use of these bundles for performance comparison between EMS systems include but are not limited to similarity in infrastructure and clinical sophistication of the prehospital services and standardized data collection.

## 6. Implications for the Future

The ultimate goal of performance indicators in EMS is to measure the true value of the system. A lot of work has been done to find the right system metrics for EMS system's evaluation. Evidence-based bundles can be good measures of the effectiveness of the system when it comes to specific clinical conditions and patient outcome. These however represent only one perspective of what good quality prehospital care means. Different stakeholders have different perspectives on quality care [44, 45]. A transition towards “Whole System Measures” defined by the Institute for Healthcare Improvement (IHI) as “balanced set of system level measures which are aligned with the Institute of Medicine's (IOM's) six dimensions of quality and are not disease or condition specific” can help overcome some of the challenges of evaluating quality in EMS [46]. Patient satisfaction with care score, rate of adverse events, incidence of occupational injuries and illnesses, and healthcare cost per capita are some examples of these whole systems measures [46]. These measures would be part of a balanced score card or measurement dashboard with specific set goals for improvement that are communicated across all levels of the EMS system from prehospital providers to leadership and policy makers. This integration of whole system measures into EMS system evaluation can help answer the question: what value is the system adding to patient care and what is the quality of the services provided?

## Figures and Tables

**Table 1:** Structure-Process-Outcome Model for EMS systems PIs.

Indicator Type	Definitions	EMS systems PI examples	Advantages	Limitations
Structure	Characteristics of the different components of the system	(i) Facilities (ii) Equipment (iii) Staffing (iv) Knowledge base of providers (v) Credentials (vi) Deployment (vii) Response times	(i) Standardized structural data allows for comparison between systems structure	(i) Indirect measure of quality (ii) Difficult to relate to outcome (iii) Problematic with EMS system design diversity

Process	Combination or sequence of steps in patient care intended to improve patient outcome	(i) Medical protocols (ii) Medication administration (iii) Transport to appropriate facility	(i) Direct measure of quality (ii) Specific input for improvement (iii) Easy to understand and to evaluate (iv) Does not require Risk adjustment (v) Easy data collection (vi) Best for technical skill evaluation (vii) Short-term evaluation	(i) Strict criteria for generalization (ii) Can become very complex with more advanced care (i.e., complex processes)

Outcome	Changes in health and well-being related to antecedent care 6 D's* (i) *Death * (ii)* Disease * (iii)* Disability * (iv)* Discomfort * (v)* Dissatisfaction * (vi) *Destitution *	(i) Out of hospital cardiac arrest survival (ii) Patient Satisfaction (iii) Improvement in pain score	(i) Easy to understand (ii) Feedback about all aspects of care provided (iii) Long-term outcomes	(i) Indirect measure of quality (ii) Requires Risk adjustment and standardization of data collection

*EMS outcomes defined by Emergency Medical Services Outcomes Project (EMSOP).

**Table 2:** Comparison of EMS clinical performance indicators.

US clinical performance indicators*						
Clinical condition	ST Elevation Myocardial infarction (STEMI)	Pulmonary Edema	Asthma	Seizure	Trauma	Cardiac arrest
Indicators or bundle elements	(1) Aspirin (2) 12 lead Electrocardio-graph (ECG) (3) Direct transport to percutaneous cardiac intervention (PCI) interval from ECG to balloon <90 minutes	(1) Nitroglycerin (2) Noninvasive positive pressure ventilation	(1) *β*_2_ agonist administration	(1) Blood Sugar measurement (2) Administration of a benzodiazepine	(1) Entrapment time <10 minutes (2) Direct transport to trauma for patients meeting criteria	(1) Response interval <5 min for basic CPR and Automated external defibrillators (AEDs)

Outcome	NNT = 15 Harm avoided: A stroke, 2nd myocardial infarction, or death	NNT = 6 Harm avoided: need for an endotracheal intubation	Not Specified	NNT = 4 Harm avoided: persistent seizure activity	NNT = 3 or 11 depending on criteria used Harm avoided: one death	NNT = 8 Harm avoided: one death

UK clinical performance indicators^#^						
Clinical condition	STEMI	Stroke/TIA	Asthma	Hypoglycemia	Trauma	Cardiac arrest

Indicators or bundle elements	(1) Aspirin (2) Nitroglycerin (3) Recording pain score (before and after treatment) (4) Pain medication (5) Transfer targets for thrombolysis/PCI	(1) Recording of Face Arm Speech Test (FAST) (2) Recording of blood sugar (3) Recording of blood pressure	(1) Recording of respiratory rate (2) Recording of Peak Expiratory Flow Rate (PEFR) (3) Recording of SpO_2_ (4) *β*_2_ agonist (5) Oxygen	(1) Recording of blood glucose before treatment (2) Recording of blood glucose after treatment (3) Recording treatment (4) Direct referral to appropriate health professional	Pilot indicators available only for patients with severe trauma (Glasgow Coma Score, GCS < 8) (1) Recording of blood pressure (2) Recording of respiratory rate (3) Recording of SpO2 (4) Recording of pupil reaction	(1) Return of Spontaneous circulation (ROSC) on arrival to hospital (2) Presence of defibrillator on scene (3) ALS provider in attendance (4) Call to scene response ≤4 min

Outcome	Improved assessment and management of STEMI with increased survival	Improved assessment and management of stroke	Improved assessment and management of asthma	Improved assessment and management of hypoglycemia	Not specified	Improved response to and survival from cardiac arrest

*Source: [20].

^#^Source: [21].
